# Student Perspectives on a Collaborative International Doctorate of Pharmacy Program

**DOI:** 10.3390/pharmacy7030085

**Published:** 2019-07-08

**Authors:** Jennifer T. Pham, Lilian M. Azzopardi, Alan H. Lau, Jennie B. Jarrett

**Affiliations:** 1 Department of Pharmacy Practice, College of Pharmacy, University of Illinois at Chicago, Chicago, IL 60612, USA; 2 Department of Pharmacy, Faculty of Medicine & Surgery, University of Malta, Msida, MSD 2080, Malta

**Keywords:** international, collaboration, PharmD, distance education

## Abstract

**Objectives:** To evaluate the educational experience and teaching methods of the collaborative Doctorate of Pharmacy (PharmD) program between the University of Malta (UM) and the University of Illinois at Chicago (UIC). **Methods:** A 41-question survey was developed to identify student demographics, satisfaction with the PharmD program and the utility of the current curricular components. Students who enrolled in the program in May 2017 were invited to participate. The survey contained open-ended, 5-point Likert, and multiple-choice type questions. The primary outcomes were the overall satisfaction and student motivations for pursuing the program. Secondary outcomes included the level of difficulty of courses, evaluation of assessment methods, and confidence in an interdisciplinary team. **Results:** Thirty-six students completed the survey (a response rate of 83.7%). The mean age was 30.1 ± 7.9 years. The majority of the students pursued the PharmD program to improve their knowledge, skills, and opportunity for obtaining a clinical position. The mean overall satisfaction of the program was 3.81 ± 1.1 (5 = very satisfied). Among the core courses, Pharmacotherapeutics had the highest overall satisfaction (4.45 ± 0.91) and level of difficulty (3.84 ± 0.51). Students felt that the tutorials/recitation case discussion sessions were the most effective teaching method (48.4%) and ranked faculties conducting case-based lectures highest for overall performance. Most students felt somewhat confident (54.8%) for participating in a multidisciplinary team. **Conclusions:** The UM/UIC PharmD Program is a unique program, utilizing a hybrid model of teaching, including distance education, to expose students to a broad and challenging curriculum in clinical pharmacy practice. Students are satisfied with this collaborative, international postgraduate PharmD program.

## 1. Introduction

Pharmacy education and the profession of pharmacy in the United States (US) have both evolved tremendously over the last five decades to meet the needs of patient care. In the United States, the American Association of Colleges of Pharmacy (AACP) mandated a Doctorate of Pharmacy (PharmD) as the only entry-level professional degree for pharmacists, beginning with the graduating class of 2006 [1]. The PharmD curriculum provides an additional year of clinical experiential education to the bachelor’s degree level program to produce pharmaceutical care experts by emphasizing a patient-centered course of study and a structure that will enable the students to develop into well-rounded practitioners.

In Europe, according to the European Union (EU) directive, pharmacy studies are of at least five years duration and adopt the Bologna education program agreement, either through an integrated master degree or through a two-cycle program. The two-cycle program consists of a bachelor’s degree after three or four years, and this is followed by a master program. The pharmacy degree program at the University of Malta consists of a two-cycle system with a 4 + 1 model. The first cycle includes a four-year full-time curriculum leading to a Bachelor of Science (Honors) in Pharmaceutical Science [B.Sc (Hons) Pharm] degree. The second cycle consists of a three-semester full-time curriculum leading to a Master of Pharmacy (MPharm) degree. The first cycle provides the graduates with an integrated approach to pharmaceutical science and pharmaceutical practice and is aimed to prepare graduates for the MPharm program. The MPharm program builds and integrates skills in pharmaceutical sciences and practice that are necessary to practice as a pharmacist in a wide variety of settings, including community pharmacy, hospital pharmacy, pharmaceutical administrative institutions, and industry and pharmaceutical regulatory sciences. Students must obtain both degrees in order to be eligible to practice pharmacy in Malta and in the European Union [2]. The Department of Pharmacy at the University of Malta identified an academic need to offer educational programs beyond the MPharm degree level as a means to facilitate the expansion of patient-centered services for pharmacists who were practicing or interested in practicing in the community, hospital, or broader pharmaceutical areas, such as in innovative medicine evaluation, pharmaceutical policy, or access to medicines.

The World Health Organization recently issued a statement that education for all healthcare professionals needs to be improved in order to meet the demand of the current healthcare needs within the global population [3]. A sufficient quantity of high-quality healthcare educators are essential to meet this global need [3]. In 2014, the University of Malta (UM) expanded their pharmacy curriculum, in collaboration with the University of Illinois at Chicago (UIC) College of Pharmacy, to include a postgraduate Doctor in Pharmacy (PharmD) degree program to support additional clinical education. Students with a pharmacy degree (bachelor or master) and a license to practice pharmacy in their respective country are eligible to apply to the UM/UIC PharmD program. As this is a level eight degree (according to the European Qualifications Framework), which is equivalent to PhD programs, this PharmD program was modified to include an additional focus on research, which is not part of current US PharmD programs.

## 2. UM/UIC PharmD Program

This collaborative, international PharmD program consists of a three-year, nine-semester, full-time curriculum. Students can either earn a Master in Advanced Clinical Pharmacy (M.Sc Adv. Clin. Pharm.) or PharmD degree, depending on the years of completion. After completion of the first year, students can opt to finish the program and receive a M.Sc Adv. Clin. Pharm. degree. Graduates with a M.Sc Adv. Clin. Pharm. degree present a specialized master with a focus on direct patient care and patient safety. Following the Doctorate of Pharmacy (PharmD) degree, graduates have a postgraduate degree equivalent to a PhD degree, with a focus on the application of pharmaceutical practice research and policy development. The practices that master and doctorate graduates are expected to follow are at higher level scales than their first degree in pharmacy. Students completing all three years will receive their PharmD degree.

The UM/UIC PharmD program combines the development and application of advanced clinical pharmacy skills and consists of didactics, clinical experiences, and applied practice research. Each semester consists of 14 weeks. The didactic courses, namely Pharmacotherapeutics I and II (two semesters), Drug Information/Statistics I and II (two semesters), Pharmacoeconomics (one semester), and Health Systems in US and Europe (one semester)] are taught by the UIC (86.1% of the courses) and UM (13.9% of the courses) faculties, taking place only during the first year of the program (Table 1). Distance education was utilized for the majority of the didactic courses, especially with Pharmacotherapeutics. Recorded (from the UIC PharmD curriculum) and live (in-person at UM and via video teleconference or in-person at UIC) lectures are paired with two- to three-hour case-based, active learning recitations that are taught either in-person or via real-time videoconferencing. The coursework also requires students to follow seminar series, participate in journal clubs, and develop scholarly reflective portfolios, all of which are provided by the UM faculty. There are five required blocks of clinical experience across pharmacy settings, including hospital pharmacy, community pharmacy, pharmacy health systems, patient-focused undergraduate student mentoring, and pharmacovigilance. First-year students spend the majority of their time in didactics (two semesters) and in two 4-week clinical experiences at the rehabilitation hospital, community pharmacy, or regulatory agency in pharmacovigilance. Third-year students spend two periods of 6-week clinical experience in an acute care setting, while the second-year students spend one. At the time of the survey, all clinical experiences took place in Malta. The research component of the curriculum consists of a clinical research dissertation (e.g., clinical service implementation/innovation), which is supervised and mentored by UM and UIC faculties. The dissertation begins during their second year and is completed during the third year.

The objective of this survey was to evaluate student perspectives on the collaborative UM/UIC PharmD program and the teaching methods provided by UIC College of Pharmacy via distance education within the UM/UIC program.

## 3. Methods

A 41-question survey was developed to assess student satisfaction with the UM/UIC PharmD program and the utility of the current curricular components provided locally and abroad. The survey questions were developed by the authors (Pham, J.T. and Jarrett, J.B.) and pilot tested by program administrators and other faculty members for readability and relevance. The survey was modified based on the feedback prior to distribution to the students. The survey contained 5-point Likert scale (1 representing extremely dissatisfied/easy and 5 representing extremely satisfied/difficult), open-ended, and multiple-choice type questions. The primary outcome was the overall satisfaction and motivations for pursuing the UM/UIC PharmD program. Secondary outcomes included the level of difficulty of courses, evaluation of assessment methods utilized within the program, and the confidence of students to participate in an interdisciplinary team.

All students enrolled in the UM/UIC PharmD program for the academic year of 2016–2017 were invited to participate in the survey. This academic year was selected as this was the first graduating class. The survey was delivered to participants via email through the Internet-based survey instrument Qualtrics^®^ in May 2017. Two reminder emails were sent to nonrespondents to encourage participation. Data collection concluded four weeks after the initial distribution. Only completed surveys were included in the analysis. However, answering every question was not required to complete the survey. The total denominators for the questions are therefore different and are noted in this report.

The results of the survey were analyzed using descriptive statistics. Continuous variables were summarized as means and standard deviations, and ordinal data as proportions. Open-ended questions regarding students’ motivations and career goals included in the survey were grouped, based on grounded theory methodology, into different categories based on their responses.

This project was approved by the Institutional Review Board at the University of Illinois at Chicago.

## 4. Results

### 4.1. Participants

A total of 43 students were identified as actively enrolled in the UM/UIC PharmD Program for the 2016–2017 academic year, qualifying them for inclusion in the study. Responses were obtained from 36 students (an 83.7% response rate). The mean age of student respondents was 30.1 years and 58.1% were female. The majority of students were Maltese (83.9%) and received their primary pharmacy degree from Malta (77.4%). Additional demographic information for these students can be found in Table 2.

### 4.2. Motivations and Career Goals

Students identified four reasons for completing the UM/UIC PharmD program: To improve their knowledge (n = 20), to increase their job opportunities (n = 11), to improve their skills (n = 7), and from the encouragement of a mentor (n = 1). Student career plans included: Clinical pharmacy practice (n = 16), regulatory affairs positions (n = 8), academia (n = 5), research (n = 2), or community practice (n = 2). Three students were still undecided in their career pursuits. The career pursuits of the majority of students (n = 20) did not change after starting the PharmD program. Of those students where their career path changed, eight were second-year and two were third-year students, noting they were more interested in clinical pharmacy practice and entrepreneurship.

### 4.3. Satisfaction

Overall, students were satisfied with the program, with a mean 5-point Likert scale score of 3.81 ± 1.12. The satisfaction of the first- and third-year classes was 4.00 ± 0 and 4.00 ± 1.04, respectively, while the mean satisfaction of second-year students was 3.60 ± 1.31. By course, Pharmacotherapeutics had the highest overall satisfaction (4.45 ± 0.91; 1 = extremely dissatisfied, 5 = extremely satisfied) as well as the highest level of difficulty (3.84 ± 0.51; 1 = extremely easy, 5 = extremely difficult). Sixty-eight percent (19/28) responded that they were most satisfied with Pharmacotherapeutics because it was taught by knowledgeable and specialized lecturers. Selected quotes by students regarding their satisfaction with the Pharmacotherapeutics course include the following: “[We] have the opportunity to be lectured by world leaders in their specific areas of expertise” and “gave up to date information and involved hands on experience with work examples”. Drug Information and Health Systems in the US and Europe were reported as the least satisfactory (35.7% of students for both course) of the core courses and the least difficult courses, with 5-point Likert scale scores of 3.27 ± 0.68 and 3.03 ± 0.65, respectively. For both courses, students noted the lack of tutorials/recitation case discussions, as well as the information being not as interesting, as reasons for their dissatisfaction. The scores for satisfaction and level of difficulty by course can be found in Table 3.

### 4.4. Teaching/Assessment Methods and Clinical Rotations

**Figure 1:**
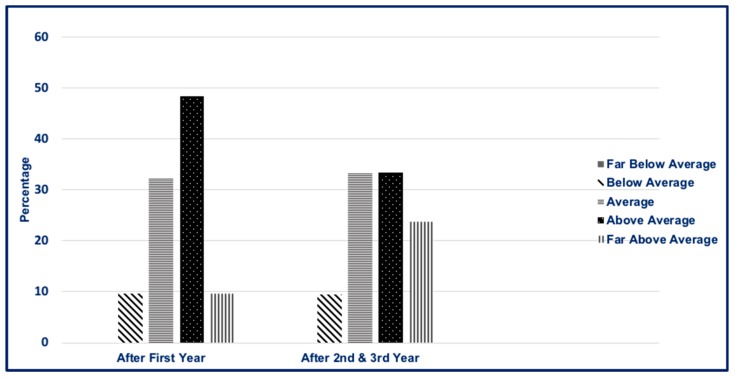
Student’s perceived preparedness for clinical rotations. ^a^ No students felt they were “far below average” for clinical rotations.

Students ranked the tutorials/recitation case discussions as the most effective teaching method (n = 15, 48.39%) compared to recorded lectures (n = 11, 35.48%) and case-based homework (n = 5, 16.13%). Faculty performance based on the type of teaching methods was rated on a 5-point Likert scale (1 = far below average, 5 = far above average). Faculties that conducted live lectures were rated highest (4.35 ± 0.70), followed by faculties that led tutorial/recitation case discussion (4.10 ± 0.93) and preceptors during rotations (3.84 ± 1.02). Students perceived a wide variety of evaluation methods to be appropriate for their knowledge and performance assessment. Scores for student perceptions of the accuracy of assessment methods are found in Table 4. Homework assignments and case-based test questions were perceived as the most suitable assessment tools. Overall, students’ perceived preparedness for clinical rotations was above average, as noted in Figure 1. Second- and third-year students were more prepared for clinical rotations (far above average) when compared to first-year students (24% compared to 10%, respectively). Students rated their level of confidence for participation in multidisciplinary teams as somewhat confident (54.8%), neither confident nor unconfident (29.03%), and somewhat unconfident (16.13%). No students rated their level of confidence as very confident or very unconfident.

## 5. Discussion

Overall, the students surveyed were satisfied with the UM/UIC PharmD program. Most students were in their early career from Malta, with no gender differences. Second-year students were slightly less satisfied with the overall program than the first- and third-year students. Students may become disenchanted with the program during the second-year of the program because of the focus on research in place of clinical knowledge and skill. Changes to the program have since been made to move one of the 6-week clinical experiences from the third year to the second year, as well as the addition of weekly formal research skill seminars during the second year to support students in the elaboration of their research proposals and the undertaking of their dissertations.

The majority of students enrolled in this program to increase their knowledge and skills, as well as increase their job opportunities within clinical pharmacy practice. These findings are comparable to a study that surveyed practicing pharmacists with bachelor’s degrees in the US to determine their interest and reason to further pursuing a PharmD program [4]. Interestingly, these US pharmacists reported the most important reasons for seeking an external PharmD degree were to increase their knowledge and skills, personal satisfaction, and career advancement [4]. While the US now requires the PharmD as the entry level degree, students enrolling in the UM/UIC PharmD program are practicing pharmacists, similar to US pharmacists holding only a bachelor’s degree. Potential students of external PharmD programs, such as the UM/UIC PharmD program, recognize the increased knowledge and skills that a postgraduate PharmD degree provides to support direct patient care practice.

Distance education is a cornerstone of UIC faculty-student interaction in the UM/UIC PharmD program, particularly within the Pharmacotherapeutics course. Interestingly, students perceived the Pharmacotherapeutics course to be the most satisfactory as well as most challenging, despite distance education. This positive response could be due to the fact that Pharmacotherapeutics utilized the most active learning methods (i.e., tutorials/recitation case discussions), which were perceived to be the most effective teaching methods by students. McLaughlin and colleagues examined the effects of a flipped classroom via video teleconferencing on student engagement, their perceptions, and academic performance [5]. A flipped classroom within this study was where information was provided prior to class to allow for more interaction. Although there was no significant difference in students’ academic performance between campuses, more students believed that learning foundational content prior to class greatly enhanced their learning during class in this distance learning situation [5]. Letassy and colleagues reported that students’ grades were higher using the team-based learning method compared to the traditional lecture-based method [6]. Team-based learning is a team-oriented teaching method that incorporates a flipped classroom and active participation in the classroom to promote self-directed learning of the course material and the application of knowledge [7]. In the UM/UIC PharmD program, team-based learning is utilized for the Pharmacotherapeutics and Drug Information and Statistics courses. Recorded lectures and case-based assignments were provided at least one week prior to tutorials/recitation case discussions to allow students the opportunity to learn foundational content and work on assignments prior to case discussions. These active learning measures can be challenging to incorporate, particularly when utilizing distance education. However, this study potentially supports the ability to implement active learning effectively from a distance through the students’ satisfaction with the course, as well as the perception of increased challenge.

To our knowledge, this is the first postgraduate collaborative PharmD program between the United States and European institutions. Currently there are many schools outside of the US that offer a PharmD degree. However, there is a wide variety of pharmacy curricula within these programs, with limitations in their clinical focus and rigor in comparison to PharmD programs in the US [8,9,10,11]. Furthermore, the quality of the programs is additionally limited by the lack of sufficiently trained clinical pharmacy faculties [8,9,10,11]. The UM/UIC PharmD program leads to a postgraduate qualification that draws on the clinical pharmacy education, training, and practice of the UIC faculty, which is comprised of active clinical pharmacists that can provide an emphasis on direct patient care in their specialty area, also integrating advanced pharmacy practice research perspectives. Clinical rotations were recently expanded for the UM/UIC program, where students can travel to UIC for clinical rotations to receive hands on experience. The information from our study highlights the capability of international collaboration, particularly with the US, to successfully educate and prepare future pharmacy clinicians. This may incite new programs to be formed to help introduce clinical pharmacy practice where it has never been before.

There are limitations in this study. The response rate and variation across classes potentially biased the results due to students encountering different aspects of the programs at different times. The program is a small program, and thus, students may have concerns regarding the anonymity of their responses. Although foreign students must satisfy an international level English language test before entering the UM/UIC PharmD program, English is not every student’s first language, and this may impose a language barrier for respondents and their understanding of the survey questions. This evaluation is limited by its cross-sectional design. We did not evaluate the students’ perception/satisfaction of the research component of the curriculum, since only a small subset of respondents (11 third-year students) would have completed the research dissertation at the time of the survey. The long-term value and efficacy of the program could be substantiated by high-level, continued job performance after completion of the program and over time.

## 6. Conclusions

A collaborative, international postgraduate PharmD program provides a unique opportunity for pharmacists to increase their knowledge and clinical skills towards direct patient care roles. This innovative program, which utilizes videoconferencing for lectures and tutorial/recitation activities, is satisfactory for student learning while providing a challenging environment for students to interact with clinical pharmacists. This type of international program allows the collaboration of institutions in need of additional faculty members to support the elevation of pharmacy education worldwide.

## Figures and Tables

**Table 1:** The University of Malta/University of Illinois at Chicago (UM/UIC) PharmD curriculum.

Education Components	First-Year	Second-Year	Third-Year
*Didactics (UM/UIC faculty)* ^1^			
Pharmacotherapeutics (6%/94%)	2 semesters		
Drug Information (27%/73%)	2 semesters		
Pharmacoeconomics (27%/73%)	1 semester		
Health Systems in US and Europe (55%/45%)	1 semester		
*Number and length of clinical experiences/rotations* ^2^	2, 4-weeks	1, 6-weeks	2, 6-weeks
*Other scholarly activities (n each year)*			
Journal club	2	2	2
Seminar series	6	2	3
*Independent Research/Dissertation*		Begins	Ends

^1^ Percentage of lectures taught by UM/UIC faculty; ^2^ At the time of the survey, all clinical rotations were completed in Malta.

**Table 2:** Demographic information for student respondents.

Characteristics	N = 32 (%)
Age (years): Mean ± SD; Range	30.1 ± 7.9; 23–50
Female gender	18 (56.3)
Malta program year	
Year 1	5 (15.6)
Year 2	16 (50.0)
Year 3	11 (34.4)
Native and country of primary pharmacy degree	
Malta	25 (78.1)
Spain	2 (6.3)
Other (Italy, India, Libya, Germany, Iraq)	5 (15.6)
Years of pharmacy practice prior to PharmD program	
Mean ± SD; Range	6.2 ± 7.6; 0–25
Previous pharmacy practice setting^a^	
Community	12 (38.7)
Hospital	8 (25.8)
Pharmaceutical regulatory affairs	7 (22.6)
Other	4 (12.9)
Previous pharmacy practice country ^a^	
Malta	26 (83.9)
Italy	1 (3.2)
India	1 (3.2)
Libya	1 (3.2)
Germany	1 (3.2)
Ireland	1 (3.2)
Students receiving financial support for PharmD program ^a^	7 (22.6)

SD = standard deviation; ^a^ Number of respondents = 31.

**Table 3:** Student satisfaction and perceived difficulty of courses.

	Overall Satisfaction ^a^	Most Satisfied	Least Satisfied	Level of Difficulty ^a^
	N = 31	N = 32	N = 28	N = 31
Courses	Mean ± SD	N (%)	N (%)	Mean ± SD
Pharmacotherapeutics	4.45 ± 0.91	28 (87.5)	1 (3.6)	3.84 ± 0.51
Drug Information & Statistics	4.19 ± 0.59	1 (3.13)	10 (35.7)	3.27 ± 0.68
Pharmacoeconomics	4.03 ± 0.69	1 (3.13)	7 (25)	3.32 ± 0.69
Health Systems in US & Europe	4.10 ± 0.78	2 (6.25)	10 (35.7)	3.03 ± 0.65

^a^ 5-point Likert scale (1 = extremely dissatisfied, 5 = extremely satisfied); N = number of respondents; SD = standard deviation.

**Table 4:** Student perceptions of the suitability of assessment methods.

Assessment Method ^a^	N (%)
*Type of Assignments*	
Homework	22 (41.51)
Final exam	19 (35.85)
Open book quiz	12 (22.64)
*Type of Exam Questions*	
Case-based	20 (34.48)
Short answer	15 (25.86)
True and false	12 (20.69)
Multiple-choice	11 (18.97)

^a^ Student could select multiple options; number of respondents = 32.

## References

[1] Buttaro M. (1992). AACP house of delegates vote: Colleges move to sole entry-level PharmD. Am. J. Hosp. Pharm..

[2] Medicines Authority. http://medicinesauthority.gov.mt/qualifiedpersons.

[3] The World Health Organization Transforming and Scaling Up Health Professionals’ Education and Training: World Health Organization Guidelines 2013. http://apps.who.int/iris/bitstream/10665/93635/1/9789241506502_eng.pdf.

[4] Joyner P.U., Pittman A.W., Campbell W.H., Dennis B.H. (1997). A needs assessment for an external doctor of pharmacy degree program. Am. J. Pharm. Educ..

[5] McLaughlin J.E., Griffin L.M., Esserman D.A., Davidson C.A., Glatt D.M., Roth M.T., Gharkholonarehe N., Mumper R.J. (2013). Pharmacy student engagement, performance, and perception in a flipped satellite classroom. Am. J. Pharm. Educ..

[6] Letassy N.A., Fugate S.E., Medina M.S., Stroup J.S., Britton M.L. (2008). Using team-based learning in an endocrine module taught across two campuses. Am. J. Pharm. Educ..

[7] Team-Based Learning Collaborative Team-based Learning Overview. http://www.teambasedlearning.org/definition.

[8] Chanakit T., Low B.Y., Wongpoowarak P., Moolasarn S., Anderson C. (2014). A survey of pharmacy education in Thailand. Am. J. Pharm. Educ..

[9] Ghilzai N.M.K., Dutta A.P. (2007). India to introduce five-year doctor of pharmacy program. Am. J. Pharm. Educ..

[10] Babar Z.U. (2005). Pharmacy education and practice in Pakistan [letter]. Am. J. Pharm. Educ..

[11] Salamzadeh J. (2004). Clinical pharmacy in Iran: where do we stand?. Iranian J. Pharm. Res..

